# Crystal structure of 1-[(4-methylbenzene)sulfonyl]pyrrolidine

**DOI:** 10.1107/S205698902000208X

**Published:** 2020-02-28

**Authors:** Brock A. Stenfors, Richard J. Staples, Shannon M. Biros, Felix N. Ngassa

**Affiliations:** a Department of Chemistry, 1 Campus Dr., Grand Valley State University, Allendale, MI 49401, USA; b Center for Crystallographic Research, Michigan State University, Department of Chemistry and Chemical Biology, East Lansing, MI 48824, USA

**Keywords:** crystal structure, sulfonamide, π–π inter­action, C—H⋯O hydrogen bonds, C—H⋯π inter­actions, polymorphism

## Abstract

The mol­ecular structure of the title compound, C_11_H_15_NO_2_S, features a sulfonamide group with S=O bond lengths of 1.4357 (16) and 1.4349 (16) Å, an S—N bond length of 1.625 (2) Å, and an S—C bond length of 1.770 (2) Å. When viewing the mol­ecule down the S—N bond, both N—C bonds of the pyrrolidine ring are oriented *gauche* to the S—C bond with torsion angles of −65.6 (2)° and 76.2 (2)°. The crystal structure features both intra- and inter­molecular C—H⋯O hydrogen bonds, as well as inter­molecular C—H⋯π and π–π inter­actions, leading to the formation of sheets parallel to the *ac* plane.

## Abstract 2

The crystal structure of a pyrrolidine-substituted *p*-toluene­sulfonamide is described. The crystal structure features both intra- and inter­molecular C—H⋯O hydrogen bonds, as well as inter­molecular C—H⋯π and π–π inter­actions, leading to the formation of sheets parallel to the *ac* plane.

## Chemical context   

Sulfonamides are of significant value in organic chemistry because of their therapeutic properties. These mol­ecules are referred to in the pharmaceutical industry as sulfa drugs. This class of drugs has been widely used in various pharmaceutical applications owing to their anti­bacterial, anti­viral, anti­malarial, anti­fungal, anti­cancer, anti­depressant, and other properties (Apaydın & Török, 2019 ▸).

**Figure 1:**
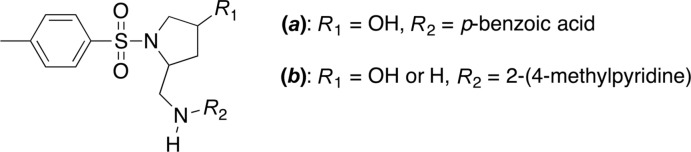
l-proline-derived 4-methyl­benzene­sulfonamide compounds that have been reported to exhibit anti-inflammatory activity against (*a*) *Trypanosoma brucei gambiense* and (*b*) to reduce brain inflammation.

N-containing heterocycles have found many uses in pharmaceutical and materials sciences, and as a result they have attracted the attention of many in the synthetic community. Numerous synthetic methods leading to N-containing heterocycles have been reported (Jiang & Ma, 2013 ▸). Notwithstanding, because of the importance of N-containing heterocycles, new and versatile synthetic methods are still desirable. The pyrrolidine-4-methyl­benzene­sulfonamide moiety is found in a variety of biologically important compounds that exhibit anti-inflammatory properties. l-proline-derived 4-methyl­benzene­sulfonamides (Fig. 1 ▸) have been reported to exhibit anti-inflammatory activity against *Trypanosoma brucei gambiense* (Ugwu *et al.*, 2018 ▸). Furthermore, these compounds can permeate the blood-brain barrier and hence can be used in treating inflammation of the brain (Ugwu *et al.*, 2017 ▸).

Generally, sulfonamides are synthesized by an analogous nucleophilic acyl-substitution reaction between an electrophile and a nucleophilic amine (Patel *et al.*, 2018 ▸). Efficient methods from the literature involve the base-catalyzed sulfonyl­ation of amines using sulfonyl halides (Yan *et al.*, 2007 ▸) or sulfonic acids (De Luca & Giacomelli, 2008 ▸) as electrophiles. The title compound, along with some related analogs, has been synthesized previously (Ohwada, *et al.*, 1998 ▸). Recently, we have discovered a more efficient method using aqueous potassium carbonate as the base. This method avoids the use of a phase-transfer catalyst by using tetra­hydro­furan as a water-miscible solvent. An increased rate of reaction and yield of sulfonamide compounds produced from a wide range of amines has been observed. These reaction conditions produced the title compound in a 91% yield, compared to the 58% yield previously reported.
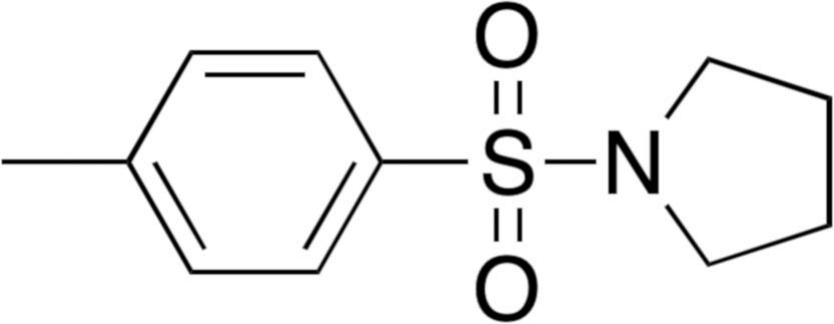


In a continuation of our research group’s ongoing inter­est in synthesizing small sulfonamide mol­ecules that mimic the structural motifs of known sulfonamide drug candidates, we synthesized the title compound, C_11_H_15_NO_2_S, and determined its crystal structure from single-crystal X-ray diffraction data.

## Structural commentary   

**Figure 2:**
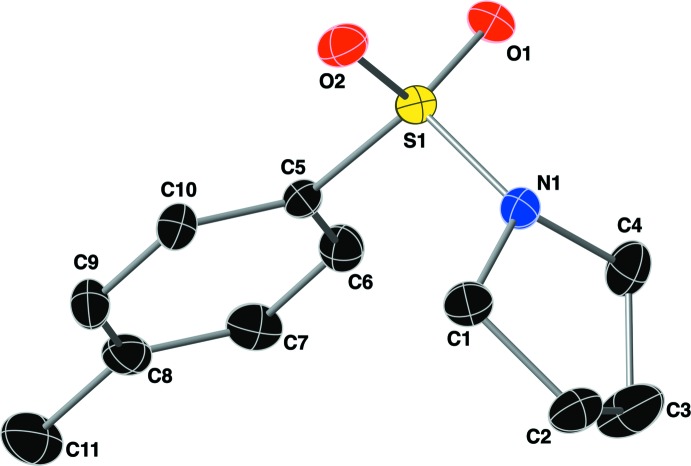
The mol­ecular structure of the title compound, with the atom-labeling scheme. Displacement ellipsoids are drawn at the 40% probability level, and all hydrogen atoms have been omitted for clarity.

The mol­ecular structure of the title compound is shown in Fig. 2 ▸. The S1=O1 and S1=O2 bond lengths are 1.4357 (16) and 1.4349 (16) Å, which is in line with known values. The S1—C5 and S1—N1 bond lengths are 1.770 (2) and 1.625 (2) Å, respectively, with an N1—S1—C5 bond angle of 107.66 (9)°. The τ_4_ descriptor for fourfold coordination around the sulfur atom, S1, is 0.94, indicating a slightly distorted tetra­hedron (ideal values are 0 for square-planar, 0.85 for trigonal–pyramidal, and 1 for tetra­hedral coordination; Yang *et al.*, 2007 ▸). Both C—N bonds of the pyrrolidine ring are oriented *gauche* to the S1—C5 bond with torsion angles C5—S1—N1—C1 = −65.62 (18)° and C5—S1—N1—C4 = 76.16 (19)°. A conformational analysis of the five-membered pyrrolidine ring pucker gives a puckering amplitude (*Q*_2_) parameter of 0.352 (3) Å and a φ_2_ parameter of 262.2 (4)°. Consequently, this ring is in a half-chair conformation with a twist along the C2—C3 bond. Lastly, an intra­molecular C—H⋯O contact (Sutor, 1958 ▸,1962 ▸,1963 ▸; Steiner, 1996 ▸) is present between H10 and O2 with an H⋯*A* distance of 2.54 Å (Table 1 ▸).

## Supra­molecular features   

**Figure 3:**
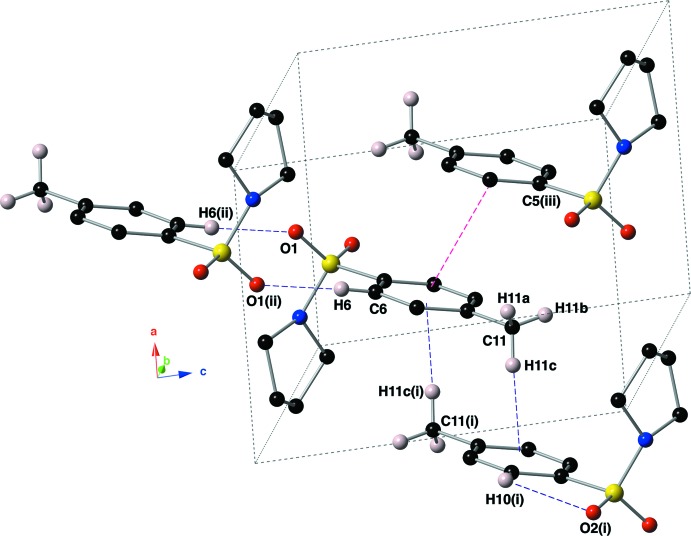
A depiction of the non-covalent inter­actions present in the crystal of the title compound using a ball-and-stick model with standard CPK colors. C—H⋯O hydrogen bonds and C—H⋯π inter­actions are shown with purple dashed lines, and π–π inter­actions are shown with magenta dashed lines. For clarity, most hydrogen atoms have been omitted and only one orientation of the intra­molecular C—H⋯O hydrogen bond is shown. [Symmetry codes: (i) −*x*, 1 − *y*, 1 − *z*; (ii) 1 − *x*, 1 − *y*, −*z*; (iii) 1 − *x*, 1 − *y*, 1 − *z*.]

**Figure 4:**
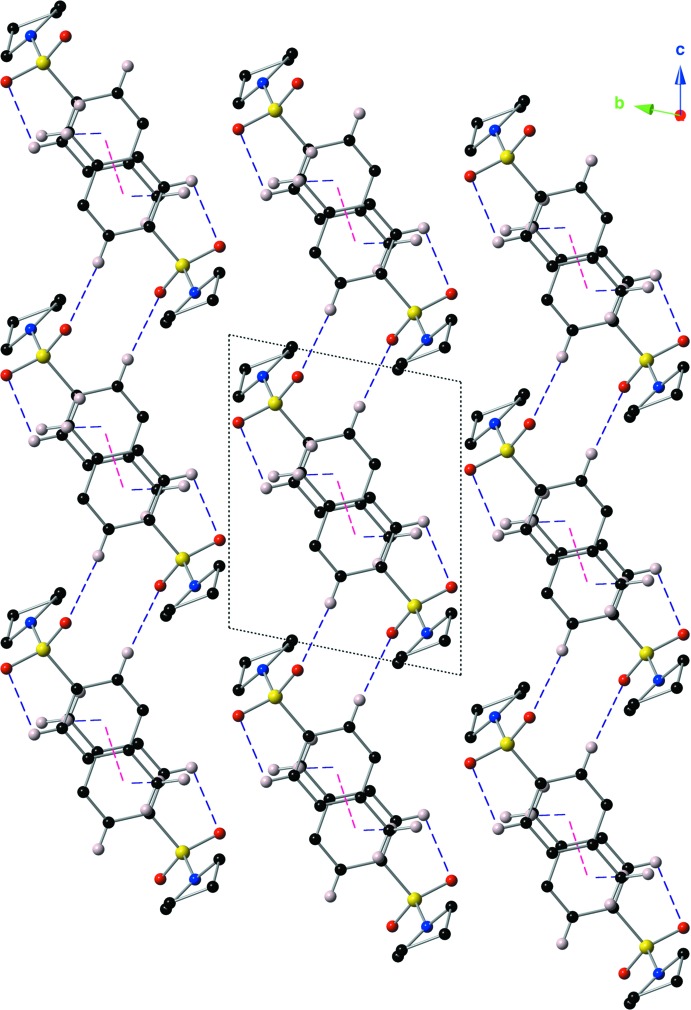
A view down the *a* axis of the crystal packing showing the supra­molecular sheets formed *via* non-covalent inter­actions. C—H⋯O hydrogen bonds and C—H⋯π inter­actions are shown with purple dashed lines, and π–π inter­actions are shown with magenta dashed lines. For clarity, only those hydrogen atoms involved in a non-covalent inter­action are shown, along with H11*A* and H11*B*.

In the crystal structure of the title compound, mol­ecules are linked by π–π inter­actions, C—H⋯O hydrogen bonds, and C—H⋯π inter­actions (Fig. 3 ▸, Table 1 ▸). The C—H⋯O hydrogen bond is formed between an aromatic C—H group (C6—H6) and one of the sulfonamide O atoms (O1). The C—H⋯π inter­action is between the methyl group (C11—H11*C*) and a symmetry-derived ring (C5–C10; symmetry code: –*x*, –*y* + 1, –*z* + 1). The π–π inter­action has a centroid-to-centroid distance of 3.8162 (15) Å with a slippage of 1.307 Å. The result of these inter­actions is the formation of sheets that lie in the *ac* plane (Fig. 4 ▸).

## Database survey   

The Cambridge Structural Database (CSD, Version 5.40, August 2019; Groom *et al.*, 2016 ▸) contains hundreds of structures that comprise a *p*-toluene­sulfonamide group bearing a pyrrolidine ring. Included in this list is another crystal-structure determination of the title compound (refcode: BABLEV; Ohwada *et al.*, 1998 ▸), which also crystallizes in the *P* 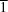 space group. Unfortunately, coordinates were not deposited for this structure at that time, so we are unable to say whether the title compound is a new packing polymorph or a new conformational polymorph. The reduced cell of BABLEV is *a* = 8.241, *b* = 2.671, *c* = 9.240 Å, *α* = 76.550, *β* = 63.800, *γ* = 87.880° with a volume of 574.55 Å^3^. The theoretical X-ray density values for each structure are similar, with 1.30 g cm^−3^ for BABLEV and 1.36 g cm^−3^ for the title compound. Thus, the more densely packed structure reported here is likely the more thermodynamically stable form.

A selection of other structures in the CSD that are closely related to the title compound are BOKPEX (Rao & Chan, 2008 ▸), GAWDAK (Chen *et al.*, 2005 ▸), VECTUT (Sherman *et al.*, 2007 ▸) and YIRCOS (Wang & Peng, 2008 ▸). These structures were chosen for comparison because they have relatively simple substituents on the pyrrolidine ring. In their paper describing the structure of GAWDAK, the authors report that this crystal also features both intra- and inter­molecular hydrogen bonds in the solid state.

## Synthesis and crystallization   

The title compound was prepared by the dropwise addition of *p*-toluene­sulfonyl chloride (1.00 g, 5.25 mmol) to a stirring mixture of pyrrolidine (0.48 ml, 5.90 mmol) and 10 ml of tetra­hydro­furan. This was followed by the dropwise addition of 0.59 *M* aqueous potassium carbonate (10 ml, 5.90 mmol) and the mixture was stirred at room temperate for 6 h. Upon acidification with 5 *M* HCl, a white precipitate was isolated by vacuum filtration to give the crude sulfonamide product. The crude product was dissolved in hot ethanol and filtered. The filtrate was transferred to a scintillation vial and crystallized upon standing for 24 h to afford colorless crystals, filtered from the mother liquor (yield 91%; m.p. 405–407 K).

## Refinement   

Crystal data, data collection and structure refinement details are summarized in Table 2 ▸. Hydrogen atoms bonded to carbon atoms were placed in calculated positions and refined as riding: C—H = 0.95–1.00 Å with *U*_iso_(H) = 1.2*U*_eq_(C) for methyl­ene groups and aromatic hydrogen atoms, and *U*_iso_(H) = 1.5*U*_eq_(C) for methyl groups.

## Supplementary Material

Crystal structure: contains datablock(s) I. DOI: 10.1107/S205698902000208X/wm5540sup1.cif

Structure factors: contains datablock(s) I. DOI: 10.1107/S205698902000208X/wm5540Isup2.hkl

CCDC reference: 1983920

Additional supporting information:  crystallographic information; 3D view; checkCIF report

## Figures and Tables

**Table 1:** Hydrogen-bond geometry (Å, °) *Cg*2 is the centroid of the C5–C10 ring.

*D*—H⋯*A*	*D*—H	H⋯*A*	*D*⋯*A*	*D*—H⋯*A*
C6—H6⋯O1^i^	0.95	2.46	3.406 (3)	174
C10—H10⋯O2	0.95	2.54	2.917 (3)	104
C11—H11*C*⋯*Cg*2^ii^	0.98	2.73	3.614 (3)	150

Symmetry codes: (i) 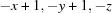; (ii) 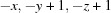.

**Table 2:** Experimental details

Crystal data	
Chemical formula	C_11_H_15_NO_2_S
*M*_r_	225.30
Crystal system, space group	Triclinic, *P* 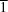
Temperature (K)	173
*a*, *b*, *c* (Å)	7.5347 (1), 8.2581 (1), 9.6157 (1)
α, β, γ (°)	77.876 (1), 86.132 (1), 69.682 (1)
*V* (Å^3^)	548.56 (1)
*Z*	2
Radiation type	Cu *K*α
μ (mm^−1^)	2.46
Crystal size (mm)	0.22 × 0.16 × 0.04

Data collection	
Diffractometer	Bruker APEXII CCD
Absorption correction	Multi-scan (*SADABS*; Krause *et al.*, 2015 ▸)
*T*_min_, *T*_max_	0.631, 0.753
No. of measured, independent and observed [*I* > 2σ(*I*)] reflections	7120, 1944, 1715
*R*_int_	0.033
(sin θ/λ)_max_ (Å^−1^)	0.603

Refinement	
*R*[*F* ^2^ > 2σ(*F* ^2^)], *wR*(*F* ^2^), *S*	0.041, 0.120, 1.09
No. of reflections	1944
No. of parameters	137
H-atom treatment	H-atom parameters constrained
Δρ_max_, Δρ_min_ (e Å^−3^)	0.53, −0.33

Computer programs: *APEX2* and *SAINT* (Bruker, 2013 ▸), *SHELXT* (Sheldrick, 2015 ▸), *OLEX2* (Dolomanov *et al.*, 2009 ▸; Bourhis *et al.*, 2015 ▸) and *CrystalMaker* (Palmer, 2007 ▸).

## supplementary crystallographic information

### Crystal data

**Table:**

C_11_H_15_NO_2_S	*Z* = 2
*M**_r_* = 225.30	*F*(000) = 240
Triclinic, *P*1	*D*_x_ = 1.364 Mg m^−^^3^
*a* = 7.5347 (1) Å	Cu *K*α radiation, λ = 1.54178 Å
*b* = 8.2581 (1) Å	Cell parameters from 3988 reflections
*c* = 9.6157 (1) Å	θ = 4.7–68.2°
α = 77.876 (1)°	µ = 2.46 mm^−^^1^
β = 86.132 (1)°	*T* = 173 K
γ = 69.682 (1)°	Plate, colourless
*V* = 548.56 (1) Å^3^	0.22 × 0.16 × 0.04 mm

### Data collection

**Table:**

Bruker APEXII CCD diffractometer	1715 reflections with *I* > 2σ(*I*)
φ and ω scans	*R*_int_ = 0.033
Absorption correction: multi-scan (*SADABS*; Krause *et al.*, 2015)	θ_max_ = 68.3°, θ_min_ = 4.7°
*T*_min_ = 0.631, *T*_max_ = 0.753	*h* = −9→9
7120 measured reflections	*k* = −9→9
1944 independent reflections	*l* = −11→11

### Refinement

**Table:**

Refinement on *F*^2^	Primary atom site location: dual
Least-squares matrix: full	Hydrogen site location: inferred from neighbouring sites
*R*[*F*^2^ > 2σ(*F*^2^)] = 0.041	H-atom parameters constrained
*wR*(*F*^2^) = 0.120	*w* = 1/[σ^2^(*F*_o_^2^) + (0.0698*P*)^2^ + 0.2666*P*] where *P* = (*F*_o_^2^ + 2*F*_c_^2^)/3
*S* = 1.09	(Δ/σ)_max_ < 0.001
1944 reflections	Δρ_max_ = 0.53 e Å^−^^3^
137 parameters	Δρ_min_ = −0.33 e Å^−^^3^
0 restraints	

### Special details

**Table:**

Geometry. All esds (except the esd in the dihedral angle between two l.s. planes) are estimated using the full covariance matrix. The cell esds are taken into account individually in the estimation of esds in distances, angles and torsion angles; correlations between esds in cell parameters are only used when they are defined by crystal symmetry. An approximate (isotropic) treatment of cell esds is used for estimating esds involving l.s. planes.

### Fractional atomic coordinates and isotropic or equivalent isotropic displacement parameters (Å^2^)

**Table:**

	*x*	*y*	*z*	*U*_iso_*/*U*_eq_	
S1	0.56522 (7)	0.19952 (7)	0.20258 (5)	0.0225 (2)	
O1	0.6545 (2)	0.2957 (2)	0.09657 (16)	0.0302 (4)	
O2	0.6789 (2)	0.0403 (2)	0.29321 (17)	0.0313 (4)	
N1	0.4193 (3)	0.1465 (2)	0.11936 (19)	0.0239 (4)	
C1	0.3110 (3)	0.0415 (3)	0.2046 (2)	0.0283 (5)	
H1A	0.263738	0.084458	0.293341	0.034*	
H1B	0.389620	−0.084784	0.228939	0.034*	
C2	0.1485 (3)	0.0702 (3)	0.1065 (3)	0.0353 (6)	
H2A	0.184331	−0.016766	0.043620	0.042*	
H2B	0.035011	0.061924	0.161429	0.042*	
C3	0.1137 (4)	0.2554 (4)	0.0214 (4)	0.0511 (8)	
H3A	0.051601	0.272209	−0.070616	0.061*	
H3B	0.032278	0.344031	0.074899	0.061*	
C4	0.3053 (4)	0.2719 (3)	−0.0018 (3)	0.0357 (6)	
H4A	0.359019	0.240275	−0.093216	0.043*	
H4B	0.299424	0.393584	−0.001822	0.043*	
C5	0.4298 (3)	0.3438 (3)	0.3132 (2)	0.0225 (5)	
C6	0.3318 (3)	0.5190 (3)	0.2538 (2)	0.0287 (5)	
H6	0.342164	0.563001	0.155156	0.034*	
C7	0.2192 (3)	0.6284 (3)	0.3400 (3)	0.0331 (6)	
H7	0.149316	0.747265	0.299159	0.040*	
C8	0.2063 (3)	0.5674 (4)	0.4859 (3)	0.0334 (6)	
C9	0.3052 (4)	0.3933 (4)	0.5423 (3)	0.0359 (6)	
H9	0.296907	0.349698	0.641258	0.043*	
C10	0.4167 (3)	0.2805 (3)	0.4575 (2)	0.0296 (5)	
H10	0.483503	0.160696	0.497955	0.035*	
C11	0.0852 (4)	0.6900 (4)	0.5788 (3)	0.0516 (8)	
H11A	0.096515	0.806829	0.547317	0.077*	
H11B	0.127699	0.643344	0.677852	0.077*	
H11C	−0.047268	0.699432	0.571301	0.077*	

### Atomic displacement parameters (Å^2^)

**Table:**

	*U*^11^	*U*^22^	*U*^33^	*U*^12^	*U*^13^	*U*^23^
S1	0.0196 (3)	0.0255 (3)	0.0225 (3)	−0.0078 (2)	−0.00028 (19)	−0.0042 (2)
O1	0.0262 (8)	0.0370 (10)	0.0289 (9)	−0.0143 (7)	0.0064 (6)	−0.0056 (7)
O2	0.0243 (8)	0.0313 (9)	0.0345 (9)	−0.0062 (7)	−0.0048 (7)	−0.0023 (7)
N1	0.0240 (9)	0.0269 (10)	0.0223 (9)	−0.0101 (8)	−0.0003 (7)	−0.0057 (7)
C1	0.0278 (11)	0.0262 (12)	0.0327 (12)	−0.0117 (9)	−0.0001 (9)	−0.0051 (9)
C2	0.0280 (12)	0.0392 (15)	0.0423 (15)	−0.0142 (10)	−0.0049 (10)	−0.0095 (11)
C3	0.0361 (15)	0.0509 (18)	0.0596 (19)	−0.0150 (13)	−0.0176 (13)	0.0090 (14)
C4	0.0395 (14)	0.0379 (14)	0.0281 (13)	−0.0140 (11)	−0.0081 (10)	0.0005 (10)
C5	0.0217 (10)	0.0269 (12)	0.0224 (11)	−0.0113 (9)	−0.0004 (8)	−0.0069 (8)
C6	0.0318 (12)	0.0295 (13)	0.0267 (12)	−0.0141 (10)	0.0011 (9)	−0.0039 (9)
C7	0.0316 (12)	0.0262 (13)	0.0443 (15)	−0.0114 (10)	0.0041 (10)	−0.0118 (10)
C8	0.0288 (12)	0.0453 (15)	0.0383 (14)	−0.0212 (11)	0.0072 (10)	−0.0215 (11)
C9	0.0370 (13)	0.0553 (17)	0.0224 (12)	−0.0224 (12)	0.0024 (10)	−0.0119 (11)
C10	0.0302 (12)	0.0361 (14)	0.0225 (11)	−0.0122 (10)	−0.0032 (9)	−0.0035 (9)
C11	0.0418 (15)	0.070 (2)	0.0624 (19)	−0.0267 (15)	0.0161 (14)	−0.0471 (17)

### Geometric parameters (Å, º)

**Table:**

S1—O1	1.4357 (16)	C4—H4B	0.9900
S1—O2	1.4349 (16)	C5—C6	1.390 (3)
S1—N1	1.6248 (18)	C5—C10	1.386 (3)
S1—C5	1.770 (2)	C6—H6	0.9500
N1—C1	1.481 (3)	C6—C7	1.382 (3)
N1—C4	1.476 (3)	C7—H7	0.9500
C1—H1A	0.9900	C7—C8	1.397 (4)
C1—H1B	0.9900	C8—C9	1.379 (4)
C1—C2	1.518 (3)	C8—C11	1.511 (3)
C2—H2A	0.9900	C9—H9	0.9500
C2—H2B	0.9900	C9—C10	1.386 (3)
C2—C3	1.517 (4)	C10—H10	0.9500
C3—H3A	0.9900	C11—H11A	0.9800
C3—H3B	0.9900	C11—H11B	0.9800
C3—C4	1.495 (4)	C11—H11C	0.9800
C4—H4A	0.9900		
			
O1—S1—N1	106.88 (9)	N1—C4—H4B	110.9
O1—S1—C5	108.04 (10)	C3—C4—H4A	110.9
O2—S1—O1	119.67 (10)	C3—C4—H4B	110.9
O2—S1—N1	106.48 (10)	H4A—C4—H4B	108.9
O2—S1—C5	107.59 (10)	C6—C5—S1	119.68 (17)
N1—S1—C5	107.66 (9)	C10—C5—S1	120.00 (18)
C1—N1—S1	118.02 (14)	C10—C5—C6	120.3 (2)
C4—N1—S1	120.69 (16)	C5—C6—H6	120.4
C4—N1—C1	110.89 (17)	C7—C6—C5	119.2 (2)
N1—C1—H1A	111.1	C7—C6—H6	120.4
N1—C1—H1B	111.1	C6—C7—H7	119.4
N1—C1—C2	103.35 (18)	C6—C7—C8	121.3 (2)
H1A—C1—H1B	109.1	C8—C7—H7	119.4
C2—C1—H1A	111.1	C7—C8—C11	120.4 (3)
C2—C1—H1B	111.1	C9—C8—C7	118.4 (2)
C1—C2—H2A	111.1	C9—C8—C11	121.2 (3)
C1—C2—H2B	111.1	C8—C9—H9	119.3
H2A—C2—H2B	109.1	C8—C9—C10	121.3 (2)
C3—C2—C1	103.2 (2)	C10—C9—H9	119.3
C3—C2—H2A	111.1	C5—C10—C9	119.5 (2)
C3—C2—H2B	111.1	C5—C10—H10	120.2
C2—C3—H3A	110.7	C9—C10—H10	120.2
C2—C3—H3B	110.7	C8—C11—H11A	109.5
H3A—C3—H3B	108.8	C8—C11—H11B	109.5
C4—C3—C2	105.2 (2)	C8—C11—H11C	109.5
C4—C3—H3A	110.7	H11A—C11—H11B	109.5
C4—C3—H3B	110.7	H11A—C11—H11C	109.5
N1—C4—C3	104.3 (2)	H11B—C11—H11C	109.5
N1—C4—H4A	110.9		
			
S1—N1—C1—C2	161.32 (16)	C1—N1—C4—C3	6.5 (3)
S1—N1—C4—C3	−137.8 (2)	C1—C2—C3—C4	36.6 (3)
S1—C5—C6—C7	176.88 (17)	C2—C3—C4—N1	−26.6 (3)
S1—C5—C10—C9	−177.89 (17)	C4—N1—C1—C2	16.0 (2)
O1—S1—N1—C1	178.52 (15)	C5—S1—N1—C1	−65.62 (18)
O1—S1—N1—C4	−39.71 (19)	C5—S1—N1—C4	76.16 (19)
O1—S1—C5—C6	38.27 (19)	C5—C6—C7—C8	1.8 (3)
O1—S1—C5—C10	−143.93 (18)	C6—C5—C10—C9	−0.1 (3)
O2—S1—N1—C1	49.53 (18)	C6—C7—C8—C9	−1.6 (3)
O2—S1—N1—C4	−168.70 (17)	C6—C7—C8—C11	178.9 (2)
O2—S1—C5—C6	168.76 (16)	C7—C8—C9—C10	0.5 (3)
O2—S1—C5—C10	−13.4 (2)	C8—C9—C10—C5	0.3 (3)
N1—S1—C5—C6	−76.84 (19)	C10—C5—C6—C7	−0.9 (3)
N1—S1—C5—C10	100.97 (19)	C11—C8—C9—C10	−179.9 (2)
N1—C1—C2—C3	−31.6 (3)		

### Hydrogen-bond geometry (Å, º)

*Cg*2 is the centroid of the C5–C10 ring.

**Table:**

*D*—H···*A*	*D*—H	H···*A*	*D*···*A*	*D*—H···*A*
C6—H6···O1^i^	0.95	2.46	3.406 (3)	174
C10—H10···O2	0.95	2.54	2.917 (3)	104
C11—H11*C*···*Cg*2^ii^	0.98	2.73	3.614 (3)	150

Symmetry codes: (i) −*x*+1, −*y*+1, −*z*; (ii) −*x*, −*y*+1, −*z*+1.

## References

[bb1] Apaydın, S. & Török, M. (2019). *Bioorg. Med. Chem. Lett.* **29**, 2042–2050. 10.1016/j.bmcl.2019.06.041 31272793

[bb2] Bourhis, L. J., Dolomanov, O. V., Gildea, R. J., Howard, J. A. K. & Puschmann, H. (2015). *Acta Cryst.* A**71**, 59–75. 10.1107/S2053273314022207 PMC4283469 25537389

[bb3] Bruker (2013). *APEX2*, *SAINT* and *SADABS*. Bruker AXS Inc. Madison, Wisconsin, USA.

[bb4] Chen, Y.-J., Xu, G., Cui, Y.-B., Huang, W. & Gou, S.-H. (2005). *Acta Cryst.* E**61**, o3571–o3573.

[bb5] De Luca, L. & Giacomelli, G. (2008). *J. Org. Chem.* **73**, 3967–3969. 10.1021/jo800424g 18393527

[bb6] Dolomanov, O. V., Bourhis, L. J., Gildea, R. J., Howard, J. A. K. & Puschmann, H. (2009). *J. Appl. Cryst.* **42**, 339–341.

[bb7] Groom, C. R., Bruno, I. J., Lightfoot, M. P. & Ward, S. C. (2016). *Acta Cryst.* B**72**, 171–179. 10.1107/S2052520616003954 PMC4822653 27048719

[bb8] Jiang, Y. & Ma, D. (2013). *Top. Organomet. Chem.* **46**, 87–118.

[bb9] Krause, L., Herbst-Irmer, R., Sheldrick, G. M. & Stalke, D. (2015). *J. Appl. Cryst.* **48**, 3–10. 10.1107/S1600576714022985 PMC4453166 26089746

[bb10] Ohwada, T., Okamoto, I., Shudo, K. & Yamaguchi, K. (1998). *Tetrahedron Lett.* **39**, 7877–7880.

[bb11] Palmer, D. (2007). *CrystalMaker*. CrystalMaker Software, Bicester, England.

[bb12] Patel, Z. S., Stevens, A. C., Bookout, E. C., Staples, R. J., Biros, S. M. & Ngassa, F. N. (2018). *Acta Cryst.* E**74**, 1126–1129. 10.1107/S2056989018010290 PMC6072992 30116576

[bb13] Rao, W. & Chan, W. H. (2008). *Chem. Eur. J.* **14**, 10486–10495. 10.1002/chem.200801242 18810730

[bb14] Sheldrick, G. M. (2015). *Acta Cryst.* A**71**, 3–8.

[bb15] Sherman, E. S., Fuller, P. H., Kasi, D. & Chemler, S. R. (2007). *J. Org. Chem.* **72**, 3896–3905. 10.1021/jo070321u PMC2590667 17428100

[bb16] Steiner, T. (1996). *Crystallogr. Rev.* **6**, 1–51.

[bb17] Sutor, D. J. (1958). *Acta Cryst.* **11**, 453–458.

[bb18] Sutor, D. J. (1962). *Nature*, **195**, 68–69.

[bb19] Sutor, D. J. (1963). *J. Chem. Soc.* pp. 1105–1110.

[bb20] Ugwu, D. I., Okoro, U. C. & Ahmad, H. (2017). *PLoS One*, **12** art. no. e0183807. 10.1371/journal.pone.0183807 PMC5602572 28922386

[bb21] Ugwu, D. I., Okoro, U. C. & Mishra, N. K. (2018). *Eur. J. Med. Chem.* **154**, 110–116. 10.1016/j.ejmech.2018.05.017 29778893

[bb22] Wang, Y.-W. & Peng, Y. (2008). *Acta Cryst.* E**64**, o56. 10.1107/S1600536807059223 PMC2915014 21200932

[bb23] Yan, J., Li, J. & Cheng, D. (2007). *Synlett*, pp. 2442–2444.

[bb24] Yang, L., Powell, D. R. & Houser, R. P. (2007). *Dalton Trans.* pp. 955–964. 10.1039/b617136b 17308676

